# Techniques for Updating Pedestrian Network Data Including Facilities and Obstructions Information for Transportation of Vulnerable People

**DOI:** 10.3390/s150924466

**Published:** 2015-09-23

**Authors:** Seula Park, Yoonsik Bang, Kiyun Yu

**Affiliations:** 1 Samsung SDS, 125 Olympic-ro 35-gil, Songpa-gu, Seoul 05510, Korea; E-Mail: seula9002@gmail.com; 2 Department of Civil and Environmental Engineering, Seoul National University, 1 Gwanak-ro, Gwanak-gu, Seoul 08826, Korea; E-Mail: bangys1004@snu.ac.kr

**Keywords:** pedestrian navigation service, transportation of vulnerable people, pedestrian network data, volunteered geographic information, facilities and obstructions

## Abstract

Demand for a Pedestrian Navigation Service (PNS) is on the rise. To provide a PNS for the transportation of vulnerable people, more detailed information of pedestrian facilities and obstructions should be included in Pedestrian Network Data (PND) used for PNS. Such data can be constructed efficiently by collecting GPS trajectories and integrating them with the existing PND. However, these two kinds of data have geometric differences and topological inconsistencies that need to be addressed. In this paper, we provide a methodology for integrating pedestrian facilities and obstructions information with an existing PND. At first we extracted the significant points from user-collected GPS trajectory by identifying the geometric difference index and attributes of each point. Then the extracted points were used to make an initial solution of the matching between the trajectory and the PND. Two geometrical algorithms were proposed and applied to reduce two kinds of errors in the matching: on dual lines and on intersections. Using the final solution for the matching, we reconstructed the node/link structure of PND including the facilities and obstructions information. Finally, performance was assessed with a test site and 79.2% of the collected data were correctly integrated with the PND.

## 1. Introduction

### 1.1. Background and Motivation

With increased use of mobile devices, the demand for a pedestrian navigation service (PNS) is on the rise. Unlike car navigation systems, pedestrian navigation may have to be used for various patterns depending on the needs of the pedestrian user. In particular, transportation of vulnerable people such as the disabled, elderly and pregnant people require more detailed guidance than other pedestrians because they have a higher likelihood of trial and error when finding their way to destinations [1]. A PNS would be particularly valuable when it is used by transportation of vulnerable people.

However, for conventional navigation there is a lack of detailed information for transportation of vulnerable people in the pedestrian network data (PND). To provide a PNS meeting the needs of the transportation of vulnerable people, detailed information on pedestrian facilities and obstructions should be included. For example, wheelchair users may access problems due to obstructions such as stairways, narrow passages, and bad paving, so that they are obliged to make detours to avoid those obstructions on the road.

Such detailed information may not be available in existing network data or other spatial databases. An alternative solution is to use volunteered geographic information (VGI), namely the collected information with help from the participation of the users. In this case, the problem is that the collected information remains separate from an existing PND. This makes it difficult to find facilities or obstructions located on the travel route. To address this problem, such information should be composed of lines or points with attributes and connected topologically with links of the PND. Then the facilities and obstructions are fully integrated with an existing PND, so that the information about them can be delivered to users at their current locations in real time.

Thus, the goal of this study is to provide a methodology for integrating information about pedestrian facilities and obstructions collected through VGI with an existing PND.

### 1.2. Related Works

Currently, there are many studies about PNS for transportation of vulnerable people. Most focus on the design of the systems or their database structures for pedestrians. Kasemsuppakorn *et al.* [2] provided a data model for a PND including detailed sidewalk information for the wheelchair navigation. Ding *et al.* [3] designed a navigation service based on GPS signals for wheelchair users and described the requirements, architectures and components of such a system. Ren *et al.* [4,5,6] studied a variety of map-matching algorithms for wheelchair navigation services. However, these studies constructed only sample datasets to verify the proposed data model and did not provide any methodology to actually construct the dataset.

In situations where the need for PNS for transportation of vulnerable people is emerging, many studies on how to build PND have been made. The conventional PND can be constructed from various sources such as satellite images, digital maps, or GPS traces. Karimi *et al.* [7] studied the approaches to generating a network map for pedestrians. They generated pedestrian network data by network buffering, collaborative mapping using GPS trace data, and image processing. The results from each process were evaluated and recommendations about a suitable approach for a given situation were provided.

Studies are in progress on utilizing GPS trajectory in the construction of PND. Based on studies about generating road line from GPS data [8,9,10], Kasemsuppakorn *et al.* [11] utilized multiple GPS data to generate a pedestrian network. Wang *et al.* [12] automatically built a road network by using the GPS traces at the intersections, junctions and U-turn nodes where pedestrians make turns. These researches demonstrated the viability of using GPS trajectory data to construct a PND. However, they assume that users move along every possible path in the test area because their purpose is to construct the initial geometry of the PND. Thus, some gaps are present where GPS traces were not collected.

On the other hand, methodologies that have used pre-existing road data were also developed. Kim *et al.* [13,14] generated a pedestrian road network for PNS by utilizing existing spatial datasets. Liu *et al.* [15] proposed the rules and logic model and attributes for a pedestrian network and proposed a connecting model with an existing vehicle network. Kim *et al.* [16] and Ballester *et al.* [17] also used the existing road networks. These approaches have advantages that the PND can be constructed relatively efficiently in a large area. However, there still lack the means for construction of network database with the information on facilities and obstructions that the transportation of vulnerable people may require.

In this study, we assume the existence of PND based on public or commercial geospatial database. Then we focus on collecting detailed information of facilities and obstructions and integrating it into the existing PND automatically. In this paper, to overcome the limitations of existing studies, we propose a methodology for matching PND with user-collected information on facilities and obstructions for transportation of vulnerable people.

This paper is set out as follows. Section 2 describes how to extract the significant points from GPS trajectory data and attributes collected through user participation, and to match geometrically with the data of the existing PND. In Section 3, we described how GPS trajectory data were collected for the experimental area and they were used to build the PND for transportation of vulnerable people. We also evaluated the results quantitatively. Section 4 discusses the conclusions and future research.

## 2. Methodology for Integrating Facilities and Obstructions Information with the Existing PND

The purpose of this study was to provide a methodology for integrating information of facilities and obstructions for transport of vulnerable people into existing PND.

To be more specific, the existing PND should be automatically integrated with the facilities and obstructions information collected through VGI. Here, the main research issue is to match the GPS-derived point or line segments to the existing PND automatically. Since the point or line segments have attributes such as facilities and obstructions, matching them to the existing PND makes it possible to automatically integrate with existing PND with information that the users can collect in the field.

There are some issues. Namely, raw GPS data have many meaningless points and some positional errors. In addition, existing PND has some inaccuracy, caused by positional error and incompleteness of the data. These discrepancies make it difficult to implement the matching process. The methods proposed in this paper focuses on solving the matching problem between the network data and the GPS trajectory.

**Figure 1:**
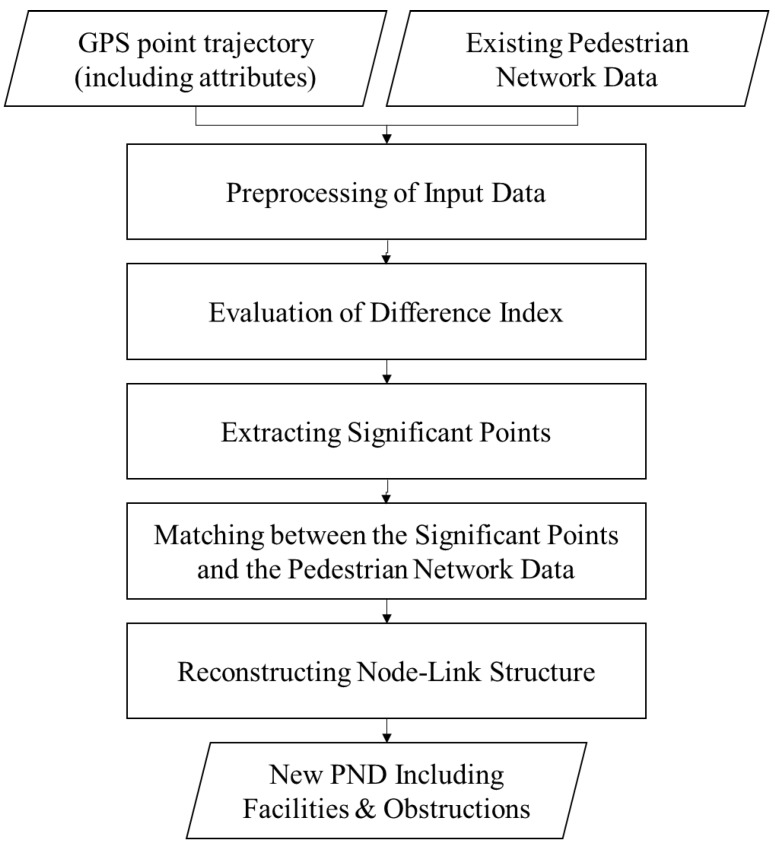
Flowchart of the process to construct the pedestrian network data (PND) for transport of vulnerable people.

Our method proposed in this paper consists of five parts, as shown in Figure 1: (1) preprocessing of the input data; (2) evaluation of difference index; (3) extracting significant points; (4) matching between the significant points and the pedestrian network data; and (5) reconstructing the node-link structure.

### 2.1. Collecting and Preprocessing of Input Data

#### 2.1.1. Collecting GPS Point Trajectory Including Attributes

Each item of facility or obstruction information is acquired along a line section that has a start point and an endpoint. In most cases, it is difficult to locate each line section on the existing PND, because a section occupies only a part of the PND. Instead, the entire GPS trajectory can be matched more accurately with the help of the contextual information. Therefore this methodology requires users to record the entire trajectory, not just parts of it, along a certain route.

To record the GPS trajectory data, a mobile device and an application for GPS data acquisition were used along the path a user moved. GPS data were collected for every second and every meter unit and as frequently as possible.

When the GPS data was acquired, we also needed to collect the attribute information, including facilities such as entrances to underpasses, overpasses, crosswalks and bridges, and obstructions such as steep slopes, narrow roads, bad paving, or raised spots. Such information was collected and saved into the attribute table attached to the corresponding GPS points at the same time as the user met the facility or obstruction.

#### 2.1.2. Defining Attributes of Facilities and Obstructions to be Collected

Categories of the facilities and obstructions to be collected in this work were selected from “Manuals for installation and management of facilities for transportation of vulnerable people” [18] and extracted types of features which can directly affect accessibility of the disabled or elderly on the outdoor walkways. Table 1 shows the specific information and code values represented in the attribute field of the GPS point data containing the facilities and obstructions information defined in this study. The additional information is composed of three fields: Road Type (shortened to “R_Type”), Wheelchair-Access Availability (“Avail”) and Obstruction (“Obs”). These represent the type of road object, wheelchair availability and type of obstruction, respectively. Table 2 describes the definitions of obstructions derived from [18].

**Table 1:** Attribute Specifications of Facilities and Obstructions Information.

Field Names	R_Type	Avail	Obs
Attributes and codes	Sidewalk: 1 Stairway: 2 Crosswalk: 3 Underpass entrance: 4 Overpass: 5 Subway entrance: 6 Building entrance: 7	Passable: 0 Difficult to pass: 1 Unable to pass: 2	Steep slope: 1 Narrow road: 2 Bad paving: 3 Raised spot: 4 Bollard: 5 Others: 6

**Table 2:** Definitions for Obstructions.

Types of Obstructions	Definitions
Steep slope	Maximum slope is higher than 1/18 (5.56%)
Narrow road	Minimum width is narrower than 1.5 m
Bad paving	Cracks or gaps between blocks are smaller than 1 cm
Raised spot	Discontinuous surface with difference in elevation larger than 2 cm
Bollard	Space between bollards is wider than 1.5 m
Others	Any other obstructions for transportation of vulnerable people

#### 2.1.3. Preprocessing of Input Data

Some outlier points do occur because the positional accuracy of GPS is lower in the beginning phase of tracking and in underground spaces, which leads to a low-intensity GPS signals, and also near tall buildings. For the collected GPS data, points located abnormally far from neighborhood points were identified as outliers and removed from the trajectory.

**Figure 2:**
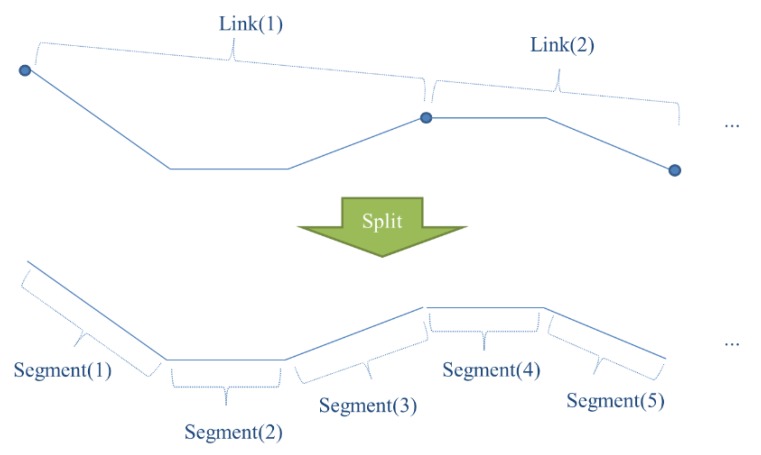
Splitting polyline objects of PND links into the unit of segments.

The methodology of this study was based on local geometric similarities of each GPS point and lines of the PND. This meant that when finding corresponding positions of each point on the GPS trajectory, each point should be found to be matched with a corresponding segment, not with a polyline object. Therefore, each polyline object of PND links was split into unit segments (see in Figure 2).

### 2.2. Evaluation of Difference Index

GPS data were acquired as point data, whereas the existing PND were line data. In this study, a Difference Index (DI) was proposed for evaluation of the similarity between heterogeneous data sets (points and lines). A lower DI value between a point and a line means that they are geometrically similar, which indicates that the point and its neighboring points are likely lying on the line.

DI values were calculated by a linear combination of the distance and the direction difference between each point and its nearby PND segments. The weight of each condition was calculated by the CRITIC (CRiteria Importance Through Intercriteria Correlation) method [19]. The process is described in detail below.

#### 2.2.1. Calculation of Geometrical Dissimilarity Variables

It is possible to calculate the DI by a linear combination of multiple geometric conditions representing the similarity measures between two spatial objects. In case of comparing the similarities between polygon objects, three criteria are generally used: position criteria calculated by the shortest distance (Euclidean distance) between the centers of objects, shape criteria calculated by perimeter-area ratio, and area criteria evaluated by overlapping two polygons [20].

In this study, the similarities between points and linear objects should be considered, instead of polygon objects. Therefore the distance (*D*) and the direction (*A*) conditions were chosen as the geometric criteria for evaluating quantitatively the similarity between a GPS point and a PND segment.

**Figure 3:**
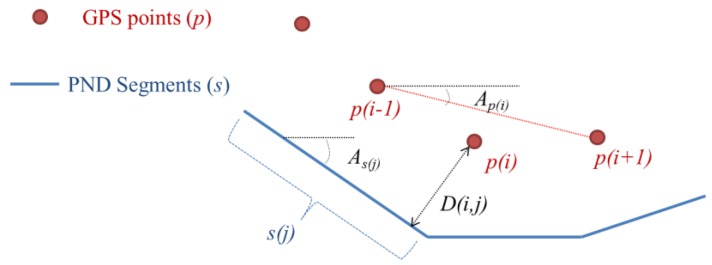
Evaluation of the distance (*D*) and the direction (*A*) conditions between GPS trajectory and PND.

At first, the distance condition means a standardized value of the shortest distance from each point to a PND segment. Since the outlier points on the GPS data were removed in the preprocessing step, it was possible to standardize the distance values by the maximum and the minimum values. In addition, the direction condition means the standardized value of an angular difference between the direction vector of a GPS point and that of a PND segment. The direction vector of a GPS point is defined as a vector that starts from its previous point and heads for its next point ($A_{p\left(i\right)}$ in Figure 3). The direction vector of a PND segment is the direction of the segment $A_{s\left(j\right)}$ in Figure 3). The angular difference in direction vectors is calculated as a tangent value of the angle, as described in Equation (2):

(1) $$D\left(i,j\right)=\sqrt{{\left(x_{p\left(i\right)}-x_{b\left(ji\right)}\right)}^{2}+{\left(y_{p\left(i\right)}-y_{b\left(ji\right)}\right)}^{2}}$$

(2) $$A\left(i,j\right)=A_{p\left(i\right)}-A_{s\left(j\right)}=\left|\frac{\left(x_{s\left(j\right),2}-x_{s\left(j\right),1}\right)\left(y_{p\left(i+1\right)}-y_{p\left(i-1\right)}\right)-\left(y_{s\left(j\right),2}-y_{s\left(j\right),1}\right)\left(x_{p\left(i+1\right)}-x_{p\left(i-1\right)}\right)}{\left(x_{s\left(j\right),2}-x_{s\left(j\right),1}\right)\left(x_{p\left(i+1\right)}-x_{p\left(i-1\right)}\right)-\left(y_{s\left(j\right),2}-y_{s\left(j\right),1}\right)\left(y_{p\left(i+1\right)}-y_{p\left(i-1\right)}\right)}\right|$$

where $\left(x_{p\left(i\right)},y_{p\left(i\right)}\right)$ are the coordinate values of the *i*-th point of the GPS trajectory; $\left(x_{b\left(ji\right)},y_{b\left(ji\right)}\right)$ are the coordinate values of the nearest point on the *j*-th segment of the PND from the *i*-th point of the GPS trajectory; $\;\left(x_{s\left(j\right),1},y_{s\left(j\right),1}\right)$ are the coordinate values of the start point of the *j*-th segment of the PND; $\;\left(x_{s\left(j\right),2},y_{s\left(j\right),2}\right)$ are the coordinate values of the endpoint of the *j*-th segment of the PND.

#### 2.2.2. Linear Combination of Variables Using the CRITIC Method

In this study, the linear combination of the distance and direction conditions were considered together, and the CRITIC method was used to reflect differences in the effects of the two conditions when the weights were calculated. The CRITIC method proposed by Diakoulaki *et al.* [19] is a method of determining the weights in consideration of the correlation and the standard deviation between each condition. It includes four steps [19]:

Step 1: standardizing each condition.
Step 2: calculating correlations between conditions to evaluate influences of conditions.
Step 3: calculating amounts of information, using the correlation coefficient and standard deviation of each condition.
Step 4: evaluating weights of conditions.

The weights of the *k*-th condition (represented as $w_{k}$) can be determined by CRITIC method, as in Equation (3):

(3) $$w_{k}=\frac{C_{k}}{\sum_{l=1}^{m}C_{l}},\;C_{k}=\sigma_{k}\overset{m}{\underset{l=1}{\sum }}\left(1-r_{kl}\right)$$

where $C_{k}$ is the “amount of information” of the *k*-th condition; $\sigma_{k}$ is the standard deviation of the *k*-th condition; $r_{kl}$ is the correlation coefficient between the *k*-th and the *l*-th conditions; m is the total number of conditions. “Amount of information” here means the unpredictability of a criterion in multi-criteria decision making (MCDM). The larger amount of information from a criterion means the higher influence to the decision making [19].

Then the Difference Index (DI) composed of the distance and direction conditions can be evaluated as in Equation (4). Here, DI(i,j) is the DI value between the *i*-th point of the GPS trajectory and the *j*-th segment of the PND:

(4) $$DI\left(i,j\right)=w_{D}D\left(i,j\right)+w_{A}A\left(i,j\right)$$

After obtaining the DI value of each GPS point with neighboring links using the above equation, a minimum DI value and the corresponding segment’s ID were added to the attribute field of the GPS points. 

### 2.3. Extracting Significant Points

From among the collected GPS point data, only the meaningful points should be selected and used to build the PND for transportation of vulnerable people. These points, the “significant points”, were extracted according to the four criteria that are described in Table 3 and Table 4 below. 

**Table 3:** Four criteria for extracting significant points from GPS point data.

No.	Description
1	Change of direction is larger than threshold
2	Difference of distances to previous and next point is larger than threshold
3	Change in attributes about facilities or obstruction occurs
4	Change of the minimum-DI segment occurs

**Table 4:** Visual examples of criteria for extracting significant points.

Criterion 1	Criterion 2	Criterion 3	Criterion 4
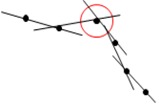	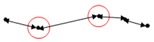	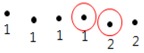	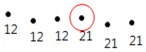

According to Kasemsuppakorn *et al*., a “significant point” is defined as a GPS point with a high probability of determining the geometry of the walking path [11]. The criteria for selecting significant points in this paper were derived from [11] but a criterion about attribute change was added. They used significant points to simplify the trajectories and construct the new network dataset, while we used them as feature points for matching user-collected GPS trajectories with the existing PND.

A change of direction is one of the most important factors in the geometrical characteristics of a trajectory. It is possible to identify the locations where a turn was made by comparing angles from the previous point and the next point. Therefore, a point can be identified as an intersection or a turning point when the change in direction is larger than the threshold. It means that the point is extracted as a significant point (Criterion 1 in Table 3 and Table 4 above).

Further, some points can deviate from the tendency in the distances between successive points. This can happen when the user temporarily stops at a facility such as a crosswalk or makes a turn at an intersection or a curve. Therefore, among the GPS points acquired, if one point has a large difference in distance from the next one and from the previous one, it is extracted as a significant point (Criterion 2).

Considering the purpose of this research for the construction of PND for transportation of vulnerable people, the attributes of facilities and obstructions in GPS points have important meanings for the output. It means that the GPS points that make changes in one or more attributes can be extracted as significant points. At each change, both the last point of the previous attribute and the first point of the next attribute are extracted (Criterion 3).

Finally, if the minimum-DI segment is changed at a point, that point would be matched with a different PND segment from the previous GPS point. The point is determined to be significant and is extracted as a significant point (Criterion 4).

### 2.4. Matching Between the Significant Points and the Pedestrian Network Data

The significant points which were extracted by the processes above should be moved onto the corresponding segments of PND links in order to be integrated with PND. By the definition of the Difference Index (DI) described in Section 2.2, it is reasonable to assume that each significant point is matched to the segment with the lowest DI value. Then the significant points are moved to the nearest locations on the corresponding segments of the PND links. Series of lines connecting the significant points in order can be regarded as the result of this map-matching process if the positional errors of GPS points lie within a reasonable range of the PND link lines.

However, this initial solution contains errors caused by the positional errors of raw GPS points and the accuracy limit of the PND. We need some correction processes for the initial solution. Previous works have tried to match GPS trajectory onto the PND [4,5,6], but their approaches are not suitable for our matching problem because the points with no information are regarded equally to the important points containing the facilities and obstruction information. Instead, we designed more proper algorithms for the error cases in the initial solution of matching between the significant points and the PND.

Error cases in the initial solution can be classified into two categories: (1) errors on dual-lined segments; and (2) errors near the intersections. In this section, the characteristics of errors are analyzed with actual cases and two geometrical processes are provided to resolve them.

#### 2.4.1. Correcting Errors on Dual-Lined Segments

**Figure 4:**
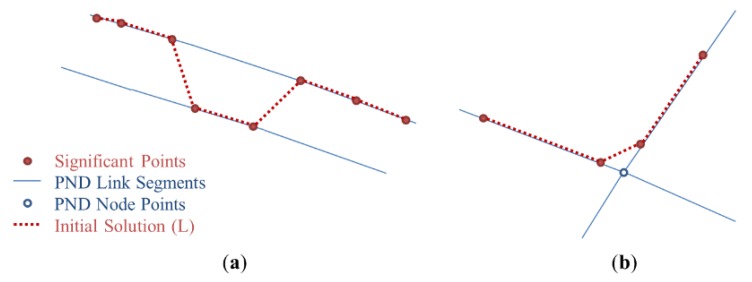
Error cases in initial solution (thin lines refer to existing PND, and thick and dotted lines refer to the initial solution of the significant points): (**a**) Example of errors on dual-lined segments; (**b**) Examples of errors near the intersections.

The first category of errors contains the errors in a GPS trajectory along a pair of parallel lines (or “dual-line”) of the PND with a distance that is less than the average positional error of GPS points. When significant points of the initial solution are moved onto the PND links consisting of a dual line, some can be moved onto the left one and others onto the right one, even though the user moved along only one of the parallel lines (Figure 4a). A geometrical process was proposed to resolve this kind of errors. A simple Algorithm (Algorithm 1: Correcting errors on dual-lined segments) of the process is described below.

**Figure 5:**
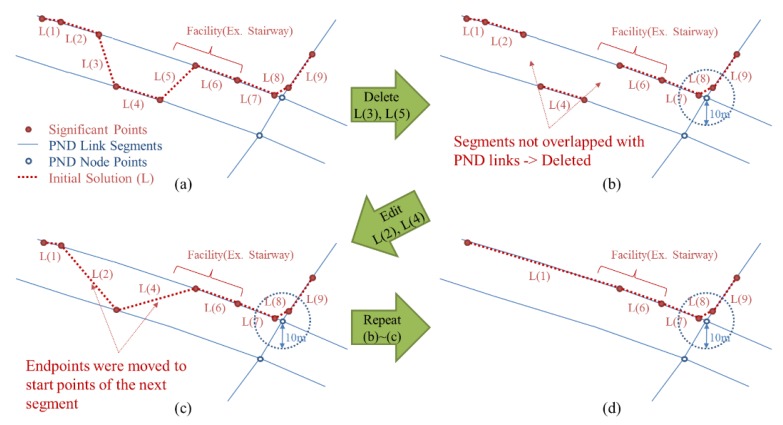
Process for correcting errors on dual-lined segments: (**a**) Initial solution after the initial matching; (**b**) Segments not overlapped with PND are deleted; (**c**) Endpoints of the initial solutions are moved to start points of the next segments; (**d**) Repeat (**a**–**c**) until there is no more segment deleted.

The significant points of the initial solution which had been distributed irregularly on both sides of the dual line now can be arranged along one of them by the process above (see in Figure 5). Which side of the dual line gathers the significant points seems to depend mostly on which side of them is nearer to the start or endpoint of the initial solution.

**Algorithm 1** Correcting errors on dual-lined segments

- Input **initial_solution**
- Input **PN_link** and **PN_node**
- Repeat:
    - for each **segment** in **initial_solution**:
        - if segment.Type is not “Crosswalk”:
             - Find the overlapped objects of **PN_link** from segment
             - if not exist overlapped objects
                  - Delete segment
        - if no deleted segment
             - Break Repeat
    - for each segment in **initial_solution**
        - if not connected with the next segment
             - Replace the endpoint with the startpoint of the next segment
- Output initial_solution


#### 2.4.2. Correcting Errors Near the Intersections

The second category of errors contains the errors in a GPS trajectory near the intersections (points where three or more segments are connected together) of the PND. When the significant points on where the trajectory made a turn are moved onto the corresponding segments of the PND, they are not projected exactly onto the intersection points because of the positional errors of GPS and the accuracy limit of the PND. Thus, the initial solution is generated as if the trajectory did not pass through the intersections and moved along shortcuts instead (Figure 4b). Another geometrical process is proposed to resolve this kind of errors. A simple Algorithm (Algorithm 2: Correcting errors near the intersections) of the process is described below.

**Algorithm**
**2** Correcting errors near the intersections
				  
- Input **initial_solution**
- Input **PN_link** and **PN_node**
- for each node in **PN_node**
    - if (number of link connected to the node) ≥ 3
         - save node in **Intersection**
- for each segment in **initial_solution**
    - Find the nearest point in **Intersection** from segment.StartPoint
    - if distance(segment.StartPoint, nearest point) ≤ 10m
         - Replace segment.StartPoint with the nearest point
    - Find the nearest point in **Intersection** from segment.EndPoint
    - if distance**(**segment.EndPoint, nearest point) ≤ 10m
         - Replace segment.EndPoint with the nearest point
- for each segment in **initial_solution**
    - if distance(segment.StartPoint, segment.EndPoint) = 0
         - Delete segment
- Output **initial_solution**


**Figure 6:**
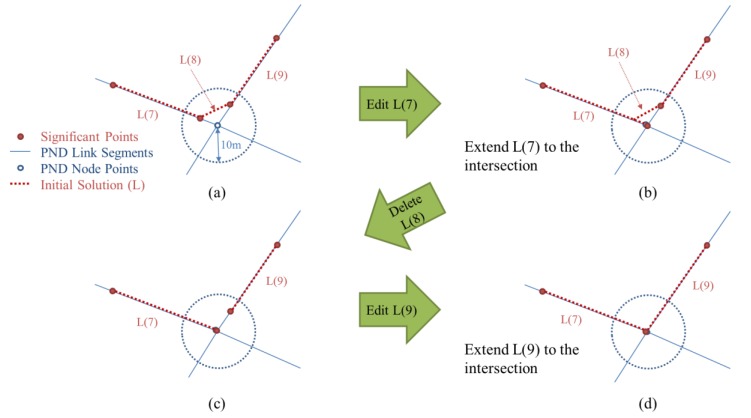
Process for correcting errors near the intersections: (**a**) Extract segments if their start or endpoints are near the intersections; (**b**) Extend the segment to the intersection; (**c**) Delete segments completely inside the buffer; (**d**) All segments are now extended to the intersection.

First, the intersection nodes are extracted from PND nodes (an intersection node is a node with three or more links connected to it, so that pedestrians can make a turn). For the initial solution that passed through the process in Section 2.4.1, we extract segments if their start or endpoints lie within a 10-m radius from the intersection nodes. The segments then are extended to the points of corresponding intersections. If the start and endpoints of a segment were both moved to the same intersection, the segment is deleted because its length becomes zero. The positional errors of the initial solution occurring near the intersections can now be resolved by the process above (see in Figure 6).

### 2.5. Reconstructing Node-Link Structure

Through the two steps of the correcting processes described above, geometrically critical errors in the initial solution were resolved. Attributes attached to the significant points should be integrated to the modified location of the initial solution.

First, the significant points containing attributes about the facilities or obstructions were extracted. They were then moved to the nearest position on the modified initial solution. Because the modified solution had its positional error reduced, it is almost coincident with the PND links. Therefore, the significant points can be moved onto the PND links accordingly.

**Figure 7:**
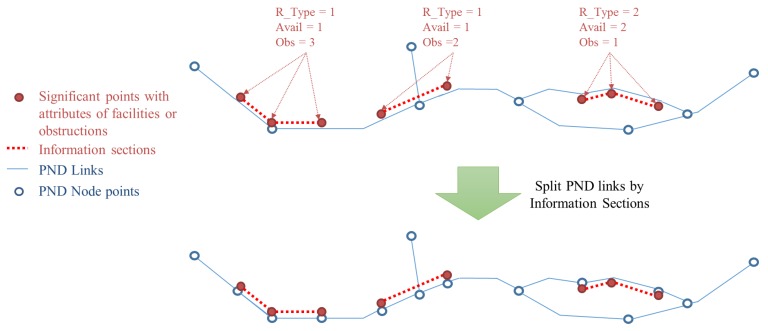
Process for splitting PND link objects at the start or endpoints of the information sections.

To find the links to be integrated with the attributes, series of points with same facility/obstruction attributes were identified, to generate polylines of “information section” data. If any two or more consecutive points have the same attributes of the three fields, Road Type (“R_Type”), Availability (“Avail”), and Obstruction (“Obs”), they are bound together and form an information section. Then the PND link objects are split at the start or endpoints of each information section. Figure 7 below describes this procedure.

**Figure 8:**
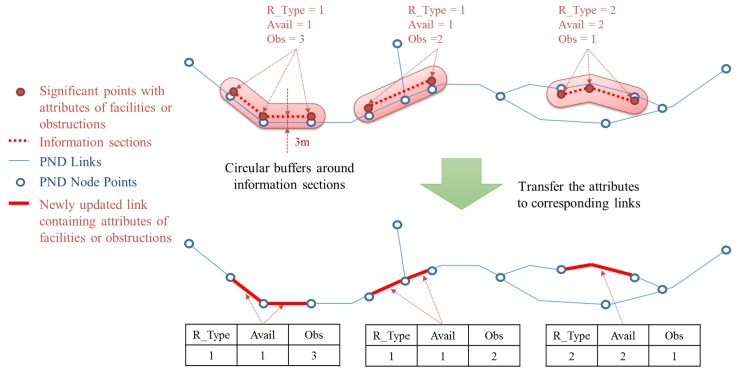
Process for extracting PND links of facilities or obstructions and transferring attributes from the significant points to the PND links.

Circular buffer polygons were generated around each information section and then used to extract the PND link objects fully contained within them. Then the attributes of the information sections were transferred to corresponding PND links (see in Figure 8). Finally, new node objects were generated at the locations where the PND links had been split, and connectivity between nodes and links were updated, so that the topology of the network data would have integrity.

The PND for transportation of vulnerable people, completed through the process above, now contains information about facilities or obstructions for them. This is not as auxiliary data; facilities and obstructions data are fully integrated into the PND. It makes possible to provide a wayfinding service for transportation of vulnerable people without using other additional information.

## 3. Experimental Result and Assessment

### 3.1. Data Used

To test the performance and check the feasibility of the methodology proposed in this study, a test area was set to collect a sample dataset by field survey. A small part (0.58 km^2^) of Macheon-dong in Seoul Metropolitan City (Republic of Korea) was set as the test area. It contains various environments for road network shapes, buildings, and open spaces. There are grid-shaped patterns and irregular patterns of road network in this area. Buildings of various heights from one to 19 stories lead to varying levels of GPS signal quality.

**Figure 9:**
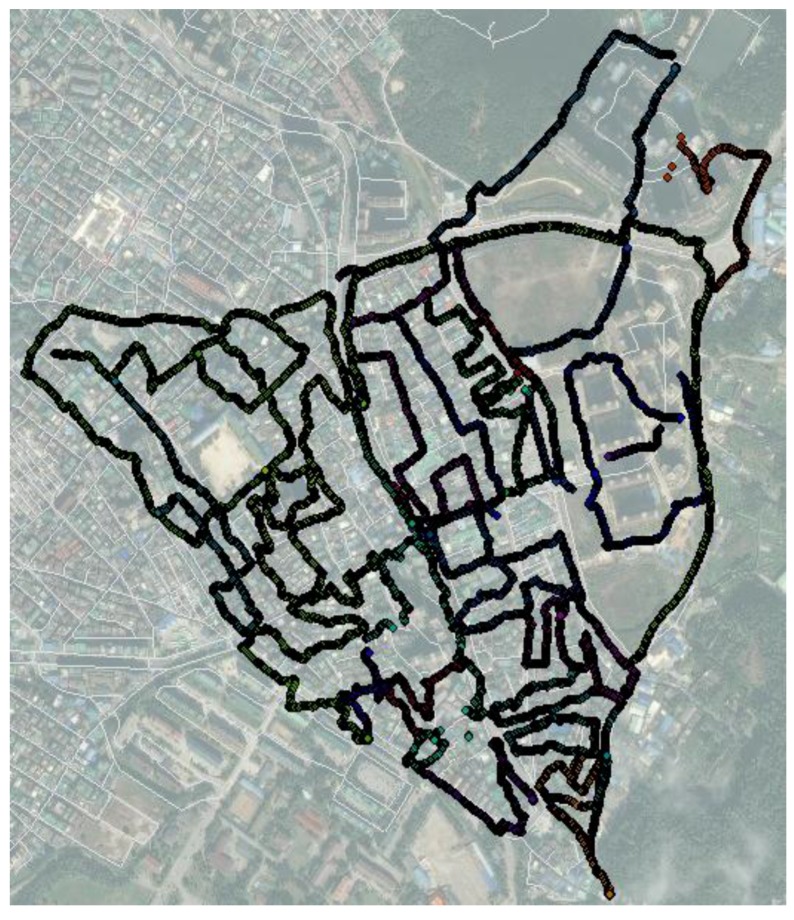
Results of field survey to collect GPS trajectories.

Field data were collected in this test area. Because we wanted the input data to be collected by users, we used smartphones to obtain GPS trajectories of the field survey. We used an application named OruxMaps, installed in smartphones with the Android OS. Positional accuracy was set to 20 m and acquisition frequency to one second and one meter. Photographs of the facilities and obstructions were used as the auxiliary data for inputting attributes in corresponding GPS points. The categories of the attributes and their definitions and codes are shown in Table 1 in Section 2.1. The recorded GPS trajectory is shown in Figure 9. Table 5 and Table 6 show examples of some GPS points, including attributes about the facility or obstruction and photos of them.

**Table 5:** Example of collected GPS points data.

Section No.	Start Time	Time Interval	End Time	R_Type	Avail	Obs
001	13:34:37	0:00:21	13:34:58	1	1	3
002	13:35:24	0:00:04	13:35:28	1	1	4
003	13:35:29	0:00:11	13:35:40	1	1	1
004	13:37:01	0:00:27	13:37:28	2	2	6
005	13:43:06	0:00:27	13:43:33	1	1	1
006	13:46:07	0:00:11	13:46:18	3	1	5
…	…	…	…	…	…	…

**Table 6:** Examples of collected data of facilities and obstructions.

Section No.	001	004	006
Photo	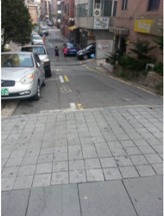	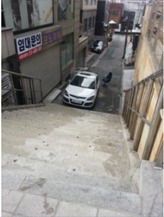	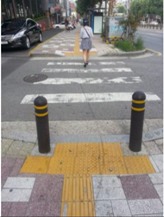

**Figure 10:**
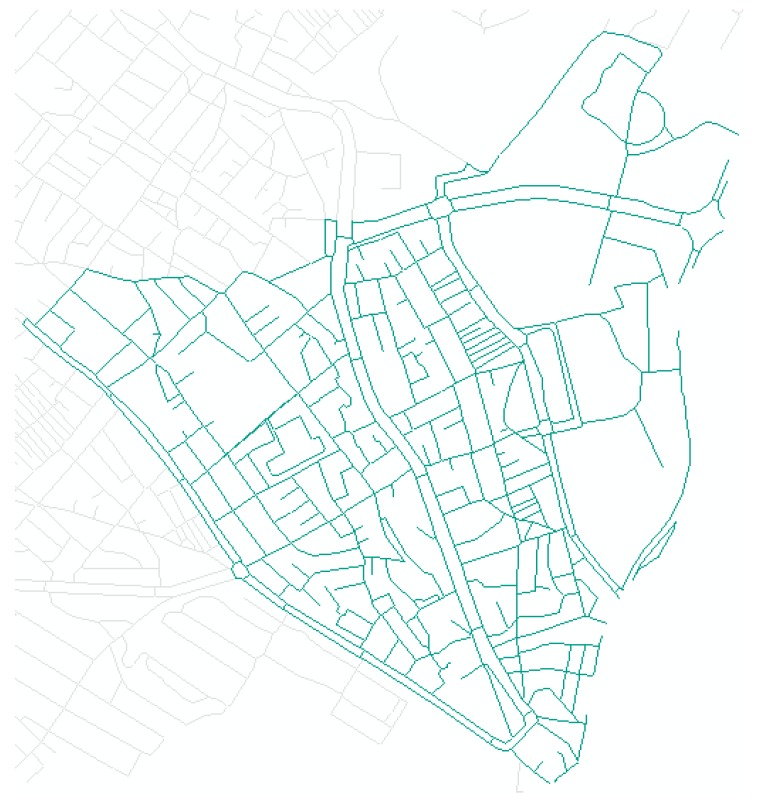
The existing PND set of the test site.

The existing PND to be integrated with the points of facilities and obstructions is shown in Figure 10. This dataset was constructed based on the digital map produced by National Geographic Information Institute (NGII) of Korea. In addition, it was constructed according to the methodology of Kim *et al.* (2015) [14], using the existing road dataset and partly manual post-processing.

Positional accuracy of the existing PND is relatively uniform while that of GPS signal differs depending on the location and circumstance. Maximum positional error of the PND is 0.7 m (horizontal), and that of the GPS trajectories collected in the test area is 18.5 m (horizontal). Although it is not appropriate to utilize GPS trajectories for adding new object to the existing PND, it is useful enough to register attributes and reorganize topology of the PND.

### 3.2. Application of the Methodology

The methodology described in Section 2 was then used to integrate the GPS trajectory datasets with the existing PND. After eliminating some outlier points, all values of distance and angle difference were calculated between every GPS point in the trajectory and its nearest link in PND. By the process described in Section 2.2, weights of geometrical dissimilarity factors between the GPS trajectories and the PND links were computed. The result of this process for sample input data is shown in Table 7.

**Table 7:** Resulted weights evaluated by CRITIC method.

Condition	Distance ($w_{D}$)	Direction Difference ($w_{A}$)
Weight	0.736	0.264

The difference index could then be evaluated by Equation (5):

(5) $$DI=w_{D}D+w_{A}A=0.736D+0.264A$$

**Figure 11:**
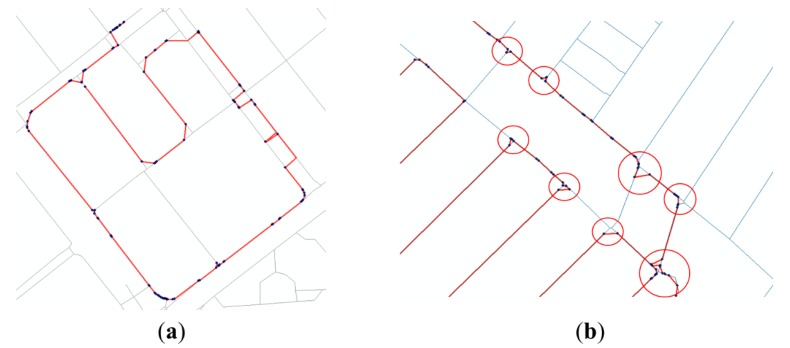
Error cases in initial solution(thin lines refer to existing PND, and thick lines refer to the initial solution of the significant points): (**a**) Example of errors on dual-lined segments; (**b**) Examples of errors near the intersections.

By the equation above, each GPS point was matched to a PND link with the lowest value of DI. Significant points were extracted by the criteria in Table 3 and moved onto the nearest positions on the corresponding PND links. The initial solution composed of the significant points is shown in Figure 11 below.

**Figure 12:**
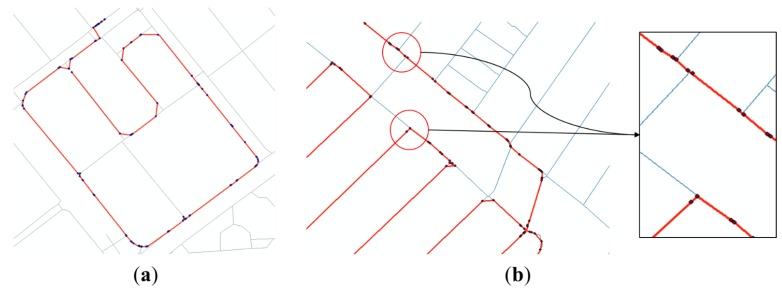
Modified solution after resolving errors (thin lines refer to existing PND, and thick lines refer to the initial solution of the significant points): (**a**) Example of errors on dual-lined segments; (**b**) Examples of errors near the intersections.

As shown in the figures above, positional errors of GPS points led to uncertainties in the matching results. Many errors occurred, especially along the dual lines or near the intersections. Applying the correction methods provided in Section 2.4 to the initial solution, the two kinds of errors described in Figure 11 were mostly resolved (see in Figure 12).

**Figure 13:**
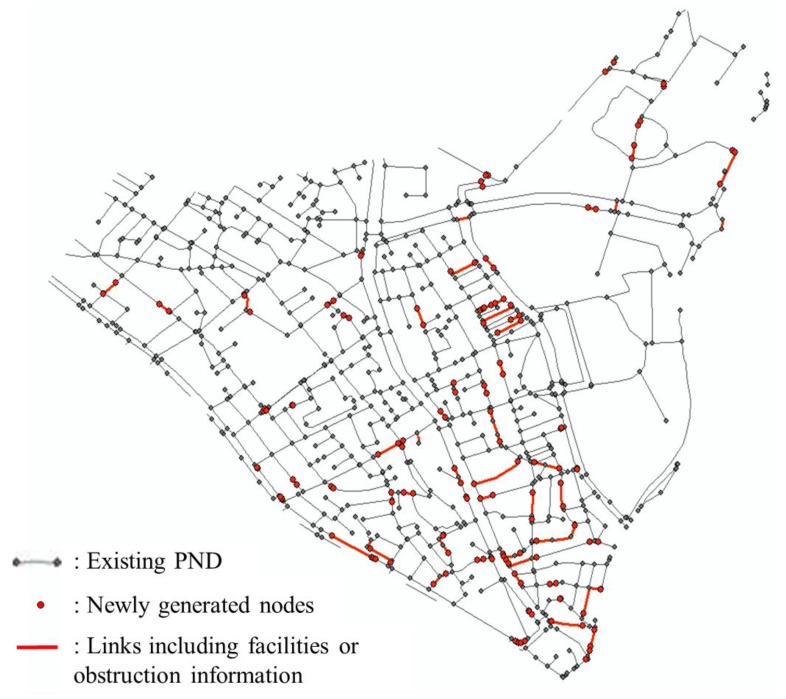
New PND of the test site integrated with the facilities and obstructions obtained by GPS trajectory of users.

Based on the result from matching with the modified solution, information of facilities and obstructions were integrated into PND and the topology relationship of links and nodes of the new PND was reconstructed accordingly. The new PND constructed on the test site is shown in Figure 13.

Through this process, 80 information sections of facilities or obstructions were registered, and 155 nodes were newly generated on positions where the links were split. The number of links was also increased by 136 in the test site.

Because the test site had many sites that were under construction when the sample dataset was collected through field survey, many information sections of bad paving were generated. Steep slopes and narrow widths were recorded in a high-density residential area. Elevators and escalators installed at subway entrances were also recorded.

### 3.3. Performance Assessment

The input data of the methodology in this paper are user-collected GPS trajectories and their attributes represent information about facilities and obstructions. Therefore, the quality of the output depends on the positional accuracy of the GPS signal, the activeness and skill of the user, the accuracy and completeness of the PND, and the performance of the method.

To assess the performance of the proposed method objectively, it is necessary to exclude factors related to quality of input data and to evaluate how much of the given input data had been reflected in the newly constructed PND containing facilities and obstructions. Therefore, performance was assessed by comparing the number of sections of facilities or obstructions collected through field survey ($N_{T}$) and the number of those generated through the proposed process ($N_{P}$). A generation rate ($R_{g}$), or a ratio of $N_{P}$ to$N_{T}$, was calculated as a measure of performance as in Equation (6):

(6) $$R_{g}=\frac{N_{P}}{N_{T}}=\frac{\text{number of sections generated by the proposed process}}{\text{number of sections collected by the field survey}}$$

When determining $N_{P}$, two or more connected information sections with exactly the same attributes were regarded and counted as one section linked together. In addition, when determining $N_{T}$, the sections included were those that were recognized by interpreting GPS trajectories and photos. The results of the performance assessment are shown in Table 8.

**Table 8:** Results of the performance assessment of the proposed methodology.

The Number of Information Sections Collected Through the Field Survey ($N_{T}$)	The Number of Information Sections Registered Through the Proposed Process ($N_{P}$)	Generation Rate ($R_{g}$, %)
101	80	79.2

**Figure 14:**
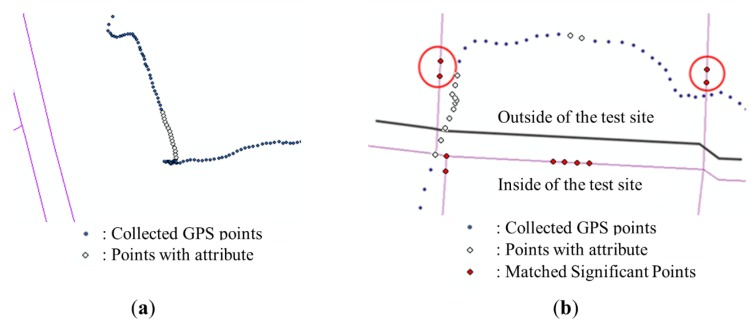
Examples of omission occurred because: (**a**) information was recorded where the PND did not have any link to be matched; (**b**) the boundary of the PND was not exactly coincident with that of the GPS data.

As Table 8 shows, 21 out of 101 information sections were omitted from the newly constructed PND through the proposed process. This omission is firstly caused by positional differences between the GPS points and the PND links. The GPS signal shows some inevitable errors caused by various factors of environment. The PND also had positional error stemming from its raw data or inherent abstractness of the network data structure. If a user recorded information at a location where the PND did not have any link to be matched, the information may not have been matched with any PND link and therefore was not integrated into the PND (Figure 14a).

The other examples of omission occurred near the boundary of the test site, because the spatial boundary of the PND did not exactly overlap with that of the GPS data. If some significant points of a facility or an obstruction were matched onto links outside of the test site (Figure 14b), those points could not be used to construct the initial solution, so the corresponding information section could not be generated. These kinds of errors were caused by the limitations of the test area, and they can be reduced if a large dataset of PND is used as input data.

## 4. Conclusions

Interest in the PNS is growing because it can be made available through the web or mobile devices, and helps people to find an optimized path while on foot. Demand for PNS for transportation of vulnerable people is also increasing as with the growing number of pedestrians using wheelchairs or other walking assistant appliances.

Researches on constructing PNDs that include sidewalks, crosswalks, passages, *etc.* have been carried out by many researchers. On the other hand, there is lack of researches for a network database for transportation of vulnerable people that contains more detailed information about the facilities and obstructions on walkways. In this paper, the methodology for constructing a PND for transportation of vulnerable people was proposed by integrating GPS trajectories of pedestrian facilities and obstructions with the existing PND.

By applying the proposed method on the sample data of the test site, a PND for transportation of vulnerable people was newly constructed. Performance was assessed by computing the generation rate ($R_{g}$) of the amount of generated data *versus* collected data. 79.2% of the collected data was integrated into the PND. 20.8% was omitted because of positional inaccuracy of the GPS signal and the inherent abstractness of the PND links. Some errors near the boundary of the test area also affected the generation rate.

By applying the methodology proposed in this research, we found that the PND which includes information about facilities and obstructions can be constructed by integrating two different kinds of datasets. Both geometry and attributes of the GPS data were used to generate the integrated network dataset. Most of the process can be done automatically, so that a large area of PND can be constructed efficiently.

Furthermore, the proposed method can be applied immediately when a single trajectory is input to the system. The other users of the navigation service can then be informed of a newly registered information about obstruction, adapting to a street environment that changes frequently. With this information, the users can search for a new route avoiding new obstructions or taking advantage of new facilities. This information may be applied to a customized navigation service, allowing use of a particular facility or obstruction based on the user’s circumstances.

Our methodology, however, depends on access to an existing PND; missing data or an inaccuracy in the PND can lead to limitations in generating data for those locations. In addition, if the positional error of the collected GPS trajectories is larger than the endurable value, wrong information sections of facilities or obstructions may be generated. These limitations will have to be overcome by future works.
